# Effect of e‐learning program for improving nurse knowledge and practice towards managing pressure injuries: A systematic review and meta‐analysis

**DOI:** 10.1002/nop2.2039

**Published:** 2023-11-30

**Authors:** Yufang Ding, Jia Qian, Yuqiong Zhou, Yibin Zhang

**Affiliations:** ^1^ Department of Operating Room, The First Affiliated Hospital, College of Medicine Zhejiang University Hangzhou Zhejiang China; ^2^ Department of Anesthesiology Hangzhou Hospital of Integrated Chinese and Western Medicine Hangzhou Zhejiang China

**Keywords:** educational program, e‐learning, nursing, pressure injury

## Abstract

**Aim:**

The aim of this review was to determine the effectiveness of the e‐learning programs for improving the knowledge and professional practices of nursing personnel in managing pressure injuries patients.

**Design:**

Systematic review and meta‐analysis.

**Methods:**

Systematic search was done in EMBASE, SCOPUS, Cochrane library, MEDLINE, Google Scholar, ScienceDirect and Clinicaltrials.gov databases until August 2022. Meta‐analysis was carried out using random‐effects model, and the results were reported as pooled standardized mean differences (SMD), or odds ratios (OR) with 95% confidence intervals (CIs).

**Results:**

Eight studies were included in the analysis. Most of the studies had higher risk of bias. The pooled SMD for knowledge score and for the classification skill were 1.40 (95%CI: 0.45–2.35; *I* ^2^ = 93.1%) and 1.75 (95%CI: 0.94–3.24; *I* ^2^ = 78.3%) respectively. The pooled OR for the classification skills was 1.75 (95%CI: 0.94–3.24; *I* ^2^ = 78.3%).

**Patient or Public Contribution:**

No patient or public contribution.

## 1 INTRODUCTION

Pressure injuries have a negative impact not only on the patients but also on the nurses and on the healthcare providers involved in the treatment (Mervis & Phillips, 2019; Mondragon & Zito, 2021). Nurses caring for patients with pressure injuries are required to work longer hours, and may feel guilt and emotional distress due to the pressure injury's onset, or the patients' delayed recovery (Bhattacharya & Mishra, 2015). Pressure injuries have also become a significant issue for hospital management that is in charge of providing reimbursements or compensation (Centers for Medicare and Medicaid Services (CMS), HHS, 2011). In addition to these issues, pressure injuries are also one of the most important indicators for assessing the quality of healthcare facility. Early detection of the condition helps in achieving faster recovery rate and reduces the unnecessary hospitalizations (Defloor & Schoonhoven, 2004; Wang et al., 2022).

For patients, nurses and healthcare facilities, prevention or early detection and proper management of pressure injuries are critical. Therefore, it is essential that nursing staff members learn how to care patients who have pressure injuries. However, previous studies indicated that the general level of competence of the nursing personnel is low (Dalvand et al., 2018; Saleh et al., 2019), and they often lack the information and the tools necessary to effectively manage a patient who has sustained a pressure injury. Therefore, it is crucial to ensure a complete awareness of the many pressure injury stages and help nurses to further develop clinical judgement, decision‐making and classification skills in order to effectively care for the pressure injury patients (Edsberg et al., 2016).

The enhancement of knowledge and skill sets among nurses is of paramount importance, and the delivery of comprehensive educational programs can promote a proactive approach in nursing practices, especially concerning pressure injury (PI) management. Traditionally, the primary method of imparting such education has centred on face‐to‐face lectures, facility‐based training or through direct integration of these components into the nursing curriculum (Eom & Jung, 2013; Niederhauser et al., 2012; Yan et al., 2022). These techniques, however, while valuable, have faced significant challenges during the COVID‐19 pandemic, emphasizing the need for more flexible, digitalized learning approaches.

In this context, e‐learning programs have been identified as promising tools for delivering educational interventions to nurses handling patients with PIs, given their ability to adapt to changing circumstances and leverage technology (Ntshwarang et al., 2021; Pokhrel & Chhetri, 2021). The majority of studies evaluating the effectiveness of such e‐learning programs have been single‐group, pre‐and post‐test design investigations, leading to limitations in the breadth and depth of the data (Kim et al., 2020; Yan et al., 2022). Since these studies tend to be influenced by natural processes, patients' characteristics and study settings, they may not provide an accurate assessment of the e‐learning programs' effectiveness. Additionally, their structure makes them unsuitable for meta‐analysis, as such a format could introduce bias and impede the ability to offer concrete recommendations to healthcare providers and administrators (Cuijpers et al., 2017).

Currently, there are limited interventional trials with comparison groups that assess the impact of e‐learning programs on the practices and knowledge of nurses managing PI patients (Beeckman et al., 2008; Bredesen et al., 2016; Morente et al., 2014; Okhovati et al., 2019). Moreover, there is a lack of pooled evidence appraising the effect of these e‐learning programs. Thus, this review seeks to address these gaps, aiming to ascertain the effectiveness of e‐learning programs in bolstering the knowledge and practices of nurses dealing with PI patients.

## 2 METHODS

### 2.1 Study design

Systematic review and meta‐analysis.

### 2.2 Study registration

PROSPERO, No. CRD42022360630.

### 2.3 Inclusion criteria (PICOS—Participants, intervention, comparison, outcomes, study design)

#### 2.3.1 Participants

Studies conducted in nurses involved in providing care for pressure injury patients or nurses working in surgical or intensive care unit (ICU) wards or home care of chronically ill patients or undergraduate nursing students undergoing training were included.

#### 2.3.2 Intervention and comparison

Studies comparing the effect of e‐learning programs for the management of pressure injuries compared to the traditional on‐campus or in‐person lecture or no training program were included.

#### 2.3.3 Outcomes

Studies reporting either the knowledge of nurses regarding pressure injuries prevention or management or practice measures (Classification skills) were included.

#### 2.3.4 Study design

Experimental trials (randomized or non‐randomized controlled trials) were eligible for inclusion. Eligible full‐text studies were included, while the case reports/series/unpublished grey literature were excluded from the study.

### 2.4 Search strategy

Systematic literature review was done by executing the search in the following databases: EMBASE, SCOPUS, Cochrane library, MEDLINE, Google Scholar and ScienceDirect. Our search strategy has combined the medical subject headings (MeSH) along with free‐text headings and suitable Boolean operators (“AND” & “OR” & “NOT”). The search terms were (“e‐learning”[All Fields] AND (“pressure ulcer”[MeSH Terms] OR (“pressure”[All Fields] AND “ulcer”[All Fields]) OR “pressure ulcer”[All Fields] OR (“pressure”[All Fields] AND “injuries”[All Fields]) OR “pressure injuries”[All Fields])). The time point of search was from inception of databases till August 2022, without any language restrictions. Database‐specific search strategy is provided in Appendix S1.

### 2.5 Study selection

During the first step in study selection process, title, keywords and abstract were screened by two independent investigators. Full‐text studies were retrieved and shortlisted. At the second step, the retrieved full texts were screened by the two investigators and those matching the eligibility criteria were included and subjected to the further analysis. ‘*Preferred Reporting Items for Systematic Reviews and Meta‐Analyses (PRISMA) checklist 2020’* was used for reporting this review (Page et al., 2021).

### 2.6 Data extraction

After finalizing the eligible full‐text articles, both investigators performed manual data extraction process using pre‐defined semi‐structured data collection form that was developed at the stage of protocol itself. Data were recorded by the first author and the entry was verified again by the second author for the correctness of data.

### 2.7 Risk of bias assessment

Two investigators were responsible for assessing the quality of the included studies. RoB2 tool (‘Cochrane risk of bias tool’) for RCTs and Cochrane risk of bias tool for non‐randomized studies were used (Sterne et al., 2016; Sterne et al., 2019). RoB2 tool is based on the following domains: randomization, deviation from intended intervention, missing data, outcome measurement and selective results reporting. RoB tool for non‐randomized studies is based on confounding, participant selection, intervention classification instead of randomization domain. All the other domains are similar to the RoB2 tool. Depending on the response, each study was identified as having low, high or some concerns with respect to the risk of bias.

### 2.8 Statistical analysis

All the analyses were performed using STATA version 14.2. For knowledge comparison, outcome was continuous in nature, and therefore, mean, standard deviation (SD) and total sample size were obtained for both groups. The pooled effect was calculated as standardized mean difference (SMD) with 95% confidence interval (CI). For binary outcomes, frequency of events and participants in intervention and control arm were entered and pooled estimate obtained as odds ratio (OR) along with the 95% CI. Random effects model with inverse variance technique was used (Cochrane Handbook for Systematic Reviews of Interventions, n.d.). Heterogeneity was evaluated by chi square of heterogeneity and *I* ^2^ statistic. Sensitivity analysis was performed to identify the single study effects on the pooled estimates. Publication bias assessment and meta‐regression could not be performed due to limitations in the number of studies.

## 3 RESULTS

### 3.1 Search results

**FIGURE 1:**
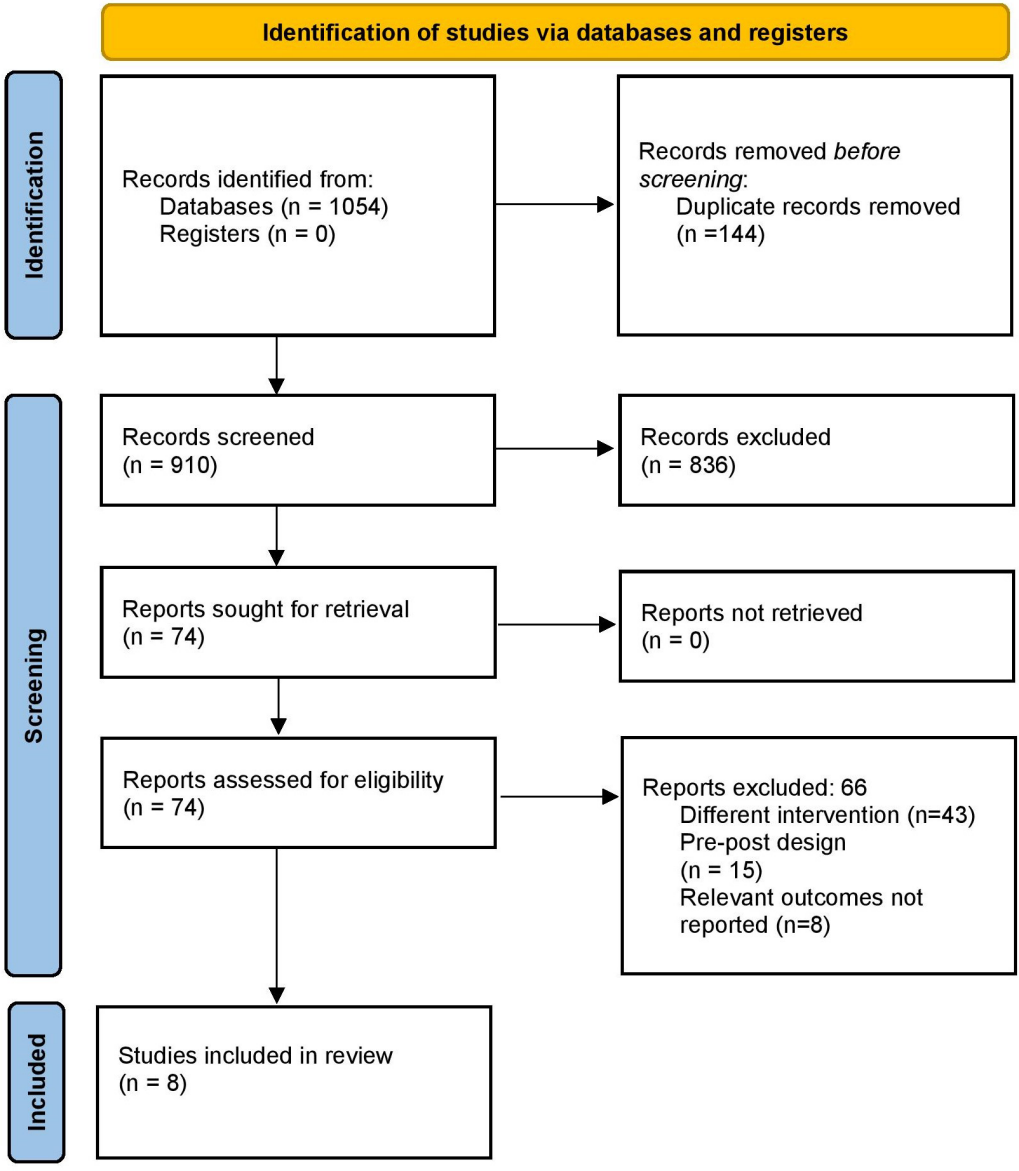
PRISMA flowchart.

A summary of the search strategy is shown in Figure 1. A total of 1054 citations were identified across all the databases during the preliminary screening. After duplicates removal and records screening, 74 full‐text studies were retrieved and subjected to the secondary screening in addition to the three articles retrieved from the bibliography of the screened articles. Finally, we included data from eight studies satisfying the inclusion criteria (Figure 1) (Beeckman et al., 2008; Bredesen et al., 2016; Cox et al., 2011; Karimian et al., 2020; Morente et al., 2014; Okhovati et al., 2019; Tubaishat, 2014; van Gaal et al., 2010).

### 3.2 Characteristics of studies included

Most studies were conducted in Iran and European countries like Norway, Belgium, Netherlands and Spain. The sample size in the intervention arm ranged between 20 and 217, while the sample size in the control arm varied between 20 and 209. Duration of the intervention varied between 45 min and 4 h. In total, four studies reported on knowledge and five studies reported on classification skills (Table 1). Most studies (six out of eight studies) had higher risk of bias as per Cochrane's tool of risk assessment for RCTs and non‐RCTs (Tables 2 and 3).

**TABLE 1:** Characteristics of the included studies (*N* = 8).

First author and year	Country	Study design	Study sample	Participants	Mean age (years)	Content of intervention	Duration of intervention	Control group	Outcomes	Measurement scale
Beeckman et al. (2008)	Belgium	RCT	I = 217 C = 209	Nursing students and qualified nurses (registered‐ and licensed nurses)	NR	Information about pressure ulcer classification	1 h	1 h lecture	Practice measures (Classification skills)	PUCLAS2
Bredesen et al. (2016)	Norway	RCT	I = 23 C = 21	Nurses working in acute care hospital wards or nursing homes	NR	Braden scale training program and PU classification	45 min	Traditional lecture	Practice measures (Classification skills)	NPUAP, photos
Cox et al. (2011)	USA	RCT	I = 20 C = 20	Medical‐surgical and critical care nurses	NR	NR	1 h	Traditional lecture	Knowledge	PUKT
Karimian et al. (2020)	Iran	RCT	I = 32 C = 35	Nurses working in ICUs	I = 33.9 C = 35.2	Prevention and treatment of bedsores	NR	NR	Knowledge	PUKT
Morente et al. (2014)	Spain	RCT	I = 30 C = 42	Undergraduate nursing students	Overall = 22.9 years	Pressure ulcer assessment and treatment	NR	Traditional on‐campus lecture	Knowledge, Practice measures (Classification skills)	PU evaluation, pictures
Okhovati et al. (2019)	Iran	Non‐randomized trial	I = 40 C = 40	Nurses working in ICUs	Overall = 34.1	Pressure ulcer definition, identification, classification	4 h	No training program	Practice measures (classification skills)	Differential Diagnostic Ability Score
Tubaishat (2014)	Jordan	Non‐randomized trial	I = 119 C = 120	Nurses working in north of Jordans	I = 31.6 C = 32.3	Pressure ulcer classification and assessment	1 h	Traditional lecture	Practice measures (classification skills)	EPUAP, photos
Van Gaal et al. (2010)	Netherlands	RCT	I = 88 C = 136	Nurses working in general wards and nursing homes	I = 36.9 C = 39	Existing Dutch guidelines for pressure ulcer prevention	150 min	No training program	Knowledge	EPUAP

*Abbreviations*: C, control; EPUAP, European Pressure Ulcer Advisory Panel; I, intervention; ICU, intensive care unit; NPUAP, National Pressure Ulcer Advisory Panel; NR, not reported; PUCLAS, Pressure Ulcer Classification; PUKT, Pieper Pressure Ulcer Knowledge test; RCT, randomized controlled trial.

**TABLE 2:** Risk of bias assessment for RCTs (*N* = 6).

Study No	Author and year	Randomization process	Deviation from intended intervention	Missing outcome data	Measurement of the outcome	Selection of the reported results	Overall
1.	Beeckman et al. (2008)	Low	Low	High	High	High	High
2.	Bredesen et al. (2016)	Some concerns	Some concerns	Low	Low	Low	Some concerns
3.	Cox et al. ( 2011)	Low	Low	Low	Low	Some concerns	Some concerns
4.	Karimian et al. (2020)	Some concerns	Some concerns	High	Low	High	High
5.	Morente et al. (2014)	Low	Low	Some concerns	Some concerns	Some concerns	High
6.	Van Gaal et al. (2010)	Low	Some concerns	High	High	Low	High

**TABLE 3:** Risk of bias assessment for non‐RCTs (*N* = 2).

S.No	Study	Selection of participants	Confounding variable	Intervention classification	Deviation from intended intervention	Incomplete outcome data	Selective reporting of outcome	Overall risk
1.	Okhovati et al. (2019)	High risk	High risk	Low risk	Unclear risk	Low risk	Unclear risk	High
2.	Tubaishat (2014)	High risk	Low risk	Low risk	Unclear risk	Low risk	Unclear risk	High

### 3.3 Nurses' knowledge regarding pressure injuries

**FIGURE 2:**
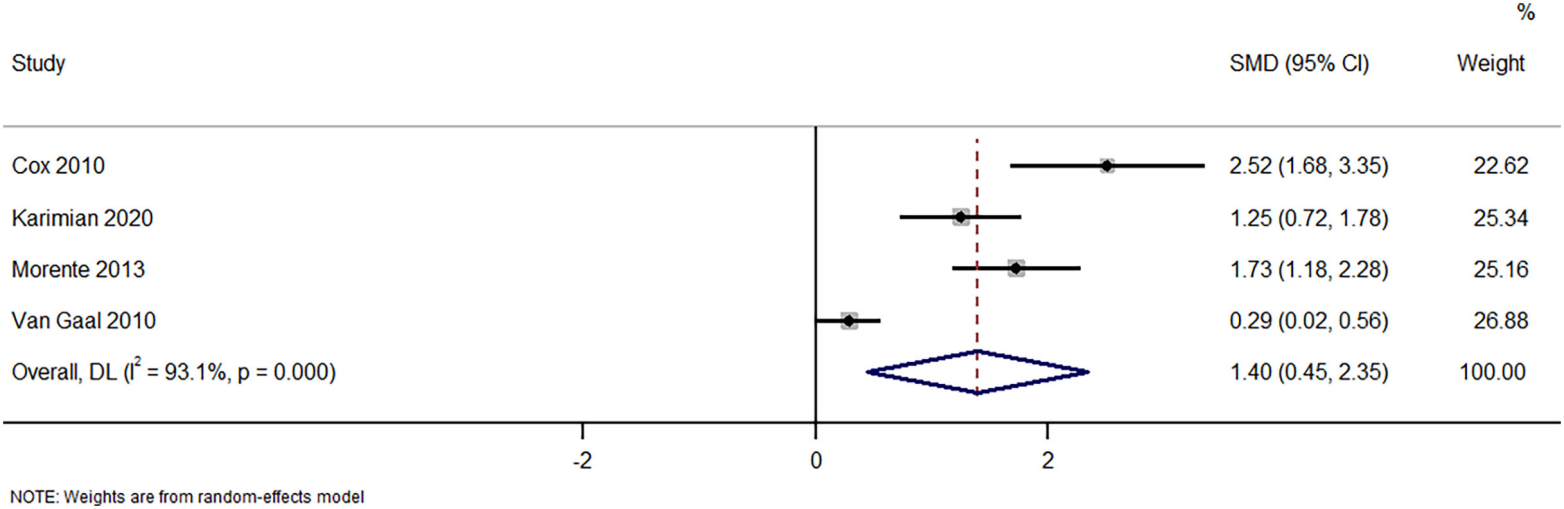
Forest plot showing the effectiveness of e‐learning program intervention in improving the knowledge about pressure injuries among nurses.

In total, four studies have reported on the effect of e‐learning programs on the knowledge of nurses about managing patients with pressure injuries. The pooled SMD was 1.40 (95%CI: 0.45–2.35; *I* ^2^ = 93.1%). This shows that nurses receiving e‐learning had significantly higher knowledge gain for managing pressure injuries compared to nurses that did not receive e‐learning, with *p*‐value of <0.004 (Figure 2). Sensitivity analysis did not show any difference in terms of magnitude or direction. This suggests that there were no single‐study effects with respect to this outcome.

### 3.4 Practice measures (classification skills)

**FIGURE 3:**
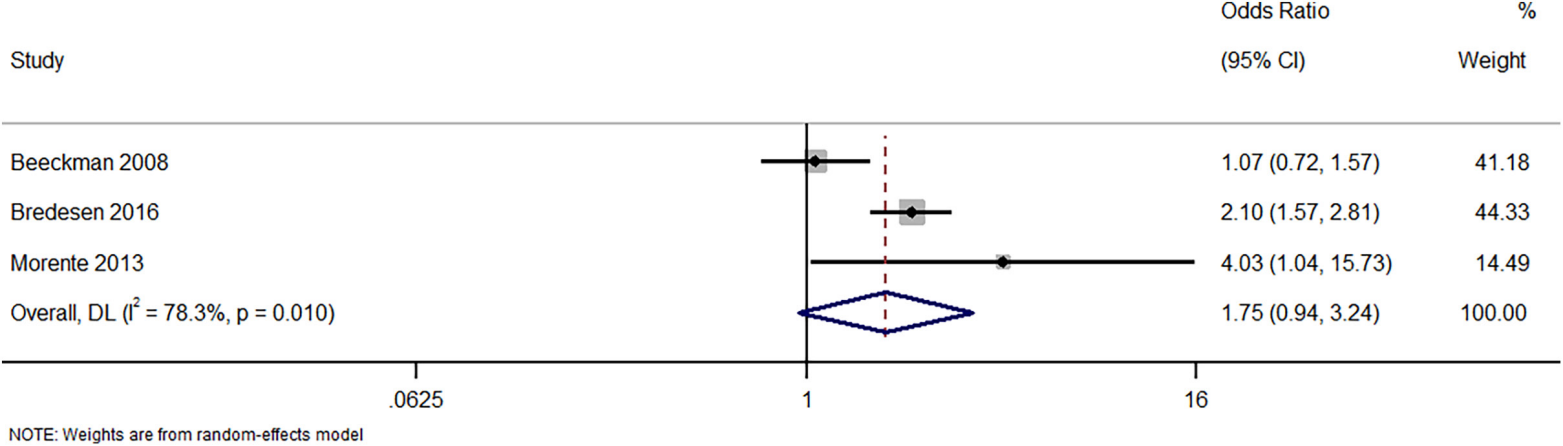
Forest plot showing the effectiveness of e‐learning program intervention in improving the classification skills (as dichotomous variable) about pressure injuries among nurses.

**FIGURE 4:**
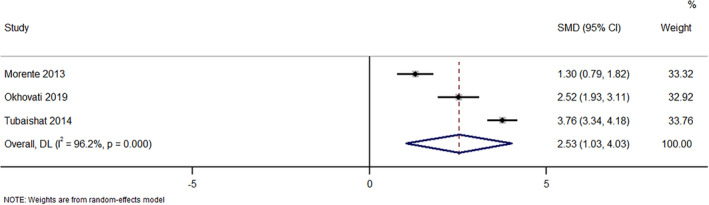
Forest plot showing the effectiveness of e‐learning program intervention in improving the classification skills (as continuous variable) about pressure injuries among nurses.

In total, five studies have reported on the effectiveness of e‐learning programs on nurse's knowledge about managing patients with pressure injuries. Of these studies, three studies have reported it as binary outcome. The pooled OR for classification skills was 1.75 (95%CI: 0.94–3.24; *I* ^2^ = 78.3%), indicating no statistically significant difference in terms of classification skills (*p* = 0.08; Figure 3). However, the pooled analysis based on studies reporting the classification skills as continuous outcome has found a statistically significant difference in classification skills in nurses receiving e‐learning programs when compared to usual or standard education programs (pooled SMD = 2.53; 95%CI: 1.03–4.03; *I* ^2^ = 96.2%; Figure 4). Sensitivity analysis did not show any difference in terms of magnitude or direction. This suggests that there were no single‐study effects with respect to this outcome.

## 4 DISCUSSION

Nurses play a pivotal role in the management of pressure injury patients. This review aimed to determine the effectiveness of e‐learning programs in improving knowledge and practices of nursing staff in managing patients with pressure injuries. A total of eight research studies were analysed, of which most were done in Iran and European nations. It is noteworthy to mention, in the context of the global pandemic, that we found no studies conducted during this period. This is surprising, given the increased reliance on virtual modes of learning and the potential for e‐learning to address the challenges posed by the pandemic in healthcare education. It is plausible that other learning priorities may have emerged during this crisis, directing the focus away from pressure injury management. Additionally, resources might have been redirected to more immediate needs, such as training for COVID‐19 case management and infection control measures. Nevertheless, the gap in research during this period highlights an area for future exploration, as the implications of the pandemic on e‐learning in pressure injury management remain largely unknown.

We found that the e‐learning programs for nurses can significantly improve their knowledge and practices in the field of pressure injury management. Though, no reviews specifically focussing on e‐learning programs were conducted before, previous reviews assessing the impact of educational program (both face‐to‐face and e‐learning) have reported that the e‐learning programs holds the edge over the face‐to‐face program in terms of knowledge and practice development in nurses (Yan et al., 2022). We also found that most studies have conducted the program for only 45 min to 2 h. This shows that e‐learning programs are very feasible and can be integrated as a part of orientation/refresher training programs for the nurses in hospitals.

Despite these findings, there are two inherent limitations associated with this study. First, immediate and residual learning effect cannot be separated and explained. Successful e‐learning modules demonstrated a significant increase in residual learning effect (Labeau et al., 2016). Since, most studies in the current review had a fixed learning time, an in‐depth analysis into this effect was not possible. Due to the design of most included studies, it was not possible to compare the learners with a longer study time against those who have invested lesser study efforts. This was again proven to be significant by Labeau et al. (2016), as the study indicated that the learners who invested more time had stronger gains in knowledge, especially residual knowledge. Albeit that this study compared progress in participants knowledge without the use of a randomized approach, it clearly illustrates that in this type of research design in which each learner with his/her own control may offer a lot of interesting information.

In addition, e‐learning programs have several other advantages in terms of lesser costs for conducting the program, wider reach and ability to cover a large group of nurses in a single program as reported by the studies included in the review (Bredesen et al., 2016; Cox et al., 2011; Karimian et al., 2020; Okhovati et al., 2019). However, standardization of content is important before proceeding with the universal implementation of e‐learning programs. Hence, a coordinated effort should be made to finalize the content and deliver the virtual training program in a more effective manner. In addition to the intervention package, there is also a need to standardize the tool used in assessing the knowledge of nurses about pressure injuries. A recent study has also developed and validated a scale to assess the knowledge level for pressure injuries, which can be utilized in the future trials as a standard tool of assessment (Botterman et al., 2022).

### 4.1 Strengths and limitations

The main strength of this review is that it is based on the comprehensive literature search in multiple databases and provides up‐to‐date evidences on the topic. We also performed sensitivity analysis and found that the review did not have any single‐study effects on the measures of association (in terms of magnitude or direction). We have included only interventional trials with independent comparison group, in contrast to the previous reviews which has also included pre‐post studies (Kim et al., 2020; Yan et al., 2022), enhancing the credibility of our evidence.

Our review also has certain limitations, and the results need to be interpreted with caution. We did not include unpublished literature, which might limit the evidence base of the review. However, inclusion of unpublished literature is highly debatable given that they are not peer‐reviewed and the reliability of the evidences are largely unknown. There was substantial statistical heterogeneity among the studies included in the review. Hence, the pooled estimate should be interpreted with high level of caution. We also could not explore the source of this heterogeneity using subgroup analysis or meta‐regression, due to the limited number of studies in the review. This variability could be due to the methodological variation between the studies in terms of setting, duration of intervention, frequency of intervention, sample size, study design (RCT or non‐RCT), etc. Finally, publication bias assessment was also not done given the limited number of studies.

### 4.2 Study implications

Despite the limitations, the current study findings support the need to develop a comprehensive standardized e‐learning programs for nurses in hospitals/clinics. Additionally, e‐learning programs that are intended to help nursing staffs are particularly useful for healthcare facilities that do not have rapid access to experts. Investing in the resource staff education is essential for optimal patient outcomes, regardless of the facility‐specific characteristics. This program needs to focus on improving the knowledge and practices of pressure injury management. Our study also highlights the need to conduct more interventional trials on the e‐learning component as an educational intervention for nurses treating pressure injuries. Further large‐scale RCTs are needed to identify the best possible e‐learning programs or curriculum to train the nurses on the management of pressure injuries and to assess short‐ and long‐term impacts of these e‐learning interventions.

## 5 CONCLUSION

Our systematic review and meta‐analysis underscore the potential of e‐learning programs in significantly enhancing the knowledge and practice of nursing staff for managing pressure injuries. Despite various e‐learning programs existing, our review emphasizes the necessity for standardization of these interventions, considering both content and measurement tools, before solidifying their effectiveness conclusively. Our findings also highlight a research gap in the field, specifically the lack of well‐designed, large‐scale randomized controlled trials (RCTs) evaluating the short‐ and long‐term impacts of e‐learning programs in this area. These future trials should aim to understand the potential nuances and variations of effectiveness in diverse settings, variations in program duration, frequency of intervention and investment of study time by the learners.

## AUTHOR CONTRIBUTIONS

Y.D. was the major contributor in writing the manuscript. J.Q. and Y.Q.Z. were contributors in the data collection and analysis. Y.B.Z. was responsible for reviewing and editing the manuscript. All authors read and approved the final manuscript.

## FUNDING INFORMATION

Not applicable.

## CONFLICT OF INTEREST STATEMENT

The authors declare that they have no competing interests.

## Supporting information


Appendix S1.

## Data Availability

The data that supports the findings of this study are available in the supplementary material of this article.
