# Supplementary material for: Effect of e‐learning program for improving nurse knowledge and practice towards managing pressure injuries: A systematic review and meta‐analysis
Source: Nurs Open. 2023 Nov 30;11(1):e2039. doi: 10.1002/nop2.2039 (PMC10697124; doi:10.1002/nop2.2039)
Supplement: Supplementary file 1 — Appendix S1. [file NOP2-11-e2039-s001.pdf]

Search Strategy:

MEDLINE (searched through PubMed search engine):

Strategy 1: ("nurse s"[All Fields] OR "nurses"[MeSH Terms] OR "nurses"[All Fields] OR "nurse"[All Fields] OR "nurses s"[All Fields]) AND ("pressure ulcer"[MeSH Terms] OR ("pressure"[All Fields] AND "ulcer"[All Fields]) OR "pressure ulcer"[All Fields] OR ("pressure"[All Fields] AND "injuries"[All Fields]) OR "pressure injuries"[All Fields]) AND "online"[All Fields] AND ("education"[MeSH Subheading] OR "education"[All Fields] OR "teaching"[All Fields] OR "teaching"[MeSH Terms] OR "teaches"[All Fields] OR "teach"[All Fields] OR "teachings"[All Fields] OR "teaching s"[All Fields])

Translations

nurse: "nurse's"[All Fields] OR "nurses"[MeSH Terms] OR "nurses"[All Fields] OR "nurse"[All Fields] OR "nurses's"[All Fields]

pressure injuries: "pressure ulcer"[MeSH Terms] OR ("pressure"[All Fields] AND "ulcer"[All Fields]) OR "pressure ulcer"[All Fields] OR ("pressure"[All Fields] AND "injuries"[All Fields]) OR "pressure injuries"[All Fields]

teaching: "education"[Subheading] OR "education"[All Fields] OR "teaching"[All Fields] OR "teaching"[MeSH Terms] OR "teaches"[All Fields] OR "teach"[All Fields] OR "teachings"[All Fields] OR "teaching's"[All Fields]

Strategy 2: ("nurse s"[All Fields] OR "nurses"[MeSH Terms] OR "nurses"[All Fields] OR "nurse"[All Fields] OR "nurses s"[All Fields]) AND ("pressure ulcer"[MeSH Terms] OR ("pressure"[All Fields] AND "ulcer"[All Fields]) OR "pressure ulcer"[All Fields] OR ("pressure"[All Fields] AND "injuries"[All Fields]) OR "pressure injuries"[All Fields]) AND ("education, distance"[MeSH Terms] OR ("education"[All Fields] AND "distance"[All

Fields]) OR "distance education"[All Fields] OR ("online"[All Fields] AND "learning"[All Fields]) OR "online learning"[All Fields])

Translations

nurse: "nurse's"[All Fields] OR "nurses"[MeSH Terms] OR "nurses"[All Fields] OR "nurse"[All Fields] OR "nurses's"[All Fields]

pressure injuries: "pressure ulcer"[MeSH Terms] OR ("pressure"[All Fields] AND "ulcer"[All Fields]) OR "pressure ulcer"[All Fields] OR ("pressure"[All Fields] AND "injuries"[All Fields]) OR "pressure injuries"[All Fields]

online learning: "education, distance"[MeSH Terms] OR ("education"[All Fields] AND "distance"[All Fields]) OR "distance education"[All Fields] OR ("online"[All Fields] AND "learning"[All Fields]) OR "online learning"[All Fields]

EMBASE:

('nurse'/exp OR 'community health nurse' OR 'community health nurses' OR 'nurse' OR 'nurse, community health' OR 'nurses' OR 'nurses, community health' OR 'nurses, public health' OR 'nursing assistance' OR 'public health nurse' OR 'public health nurses') AND ('education'/exp OR 'child education' OR 'college admission test' OR 'education' OR 'education service' OR 'education, nonprofessional' OR 'educational measurement' OR 'intellectual training' OR 'internship, nonmedical' OR 'perceptorship' OR 'preceptorship' OR 'self-evaluation programmes' OR 'self-evaluation programs' OR 'training support') AND ('decubitus'/exp OR 'bed sore' OR 'bedsore' OR 'decubital ulcer' OR 'decubitus' OR 'decubitus ulcer' OR 'decubitus ulceration' OR 'decubitus ulcers' OR 'decubitus ulcus' OR 'decubus ulcer' OR 'pressure injury' OR 'pressure sore' OR 'pressure ulcer' OR 'sore, pressure' OR 'ulcer, pressure' OR 'ulcus decubitus')

Cochrane library:

(("e-learning" OR "online learning" OR "digital learning") AND ("nursing" OR "nurse" OR "nurses") AND ("pressure injury" OR "pressure ulcer" OR "bedsore")) AND ("knowledge" OR "education" OR "training" OR "learning")

Scopus:

online AND learning AND nurse AND pressure AND injury AND (LIMIT-TO (DOCTYPE , "ar")) AND (LIMIT-TO (EXACTKEYWORD , "Human")) AND (LIMIT-TO (LANGUAGE , "English")) AND (LIMIT-TO (SRCTYPE , "j"))
